# Normal-weight central obesity: implications for diabetes mellitus

**DOI:** 10.3389/fnut.2023.1239493

**Published:** 2023-09-22

**Authors:** Xueshan Jin, Jiajun Liu, Qiuyu Cao, Jiehua Lin, Guangfu Wu, Longhui Liu, Shan Jiang, Xin Zhou, Zhiqiang Li, Aicheng Yang

**Affiliations:** ^1^Nephropathy Center, The Affiliated Jiangmen TCM Hospital of Jinan University, Jiangmen, China; ^2^First Clinical Medical College, Guangzhou University of Chinese Medicine, Guangzhou, China

**Keywords:** normal-weight central obesity, body mass index, waist circumference, diabetes mellitus, National Health and Nutrition Examination Survey

## Abstract

**Background:**

Current guidelines for obesity prevention and control focus on body mass index (BMI) and rarely address central obesity. Few studies have been conducted on the association between normal-weight central obesity and the risk of diabetes mellitus (DM).

**Methods:**

26,825 participants from the National Health and Nutrition Examination Survey (NHANES) were included in our study. A weighted multivariate logistic regression model was used to analyze the relationship between different obesity patterns and the risk of DM.

**Results:**

Our results suggest that normal-weight central obesity is associated with an increased risk of DM (OR: 2.37, 95% CI: 1.75–3.23) compared with normal-weight participants without central obesity. When stratified by sex, men with normal-weight central obesity, obesity and central obesity were found to have a similar risk of DM (OR: 3.83, 95% CI: 2.10–5.97; OR: 4.20, 95% CI: 3.48–5.08, respectively) and a higher risk than all other types of obesity, including men who were overweight with no central obesity (OR: 1.21, 95% CI: 0.96–1.51) and obese with no central obesity (OR: 0.53, 95% CI: 0.30–0.91).

**Conclusion:**

Our results highlight the need for more attention in people with central obesity, even if they have a normal BMI.

## 1. Introduction

Over the past half century, overweight and obesity have increased globally, with the prevalence of obesity increasing from 3.2 to 10.8% in men and from 6.4 to 14.9% in women (1). Excess fat can increase the incidence of metabolic diseases, however, for a given amount of fat, the risk of metabolic diseases varies greatly among different populations. For example, the risk of cardiometabolic disease was unexpectedly increased in people with metabolically unhealthy normal weight (2–4), compared to people with metabolically healthy normal weight, and only modestly increased in people with metabolically healthy obesity (2, 4–7). The characteristic fat distribution of metabolically unhealthy normal weight people is high visceral fat content, while metabolically healthy obese people are low visceral fat content (8, 9). Abdominal obesity can reflect the content of visceral fat and is one of the important phenotypes of unhealthy metabolism (10, 11).

Diabetes mellitus (DM) is one of the major diseases of global concern, affecting 422 million people and directly causing 1.5 million deaths each year (12, 13). With the acceleration of aging, the public health budget expenditure caused by DM increases year by year. Therefore, early identification of DM is of great significance in the field of public health and clinical medicine. Many indicators have been developed to predict the occurrence of DM. Anthropometric parameters have been widely reported in recent years because they are easy to measure, non-invasive, and inexpensive (14–16). BMI (defined as general obesity) and waist circumference (defined as central obesity) are important indicators for measuring obesity, and their increase is associated with a higher risk of DM (17). However, a recent study has shown that obesity as defined by BMI is inversely associated with the occurrence of DM in men (18). Another study reported similar results that insulin resistance (an early manifestation of DM) was associated with abdominal obesity, but not general obesity (19). Although BMI is a standard measure in the clinical assessment of obesity, it does not distinguish body shape or the accumulation of fatty tissue (20). Even in people with a normal BMI (18.5–24.9 kg/m^2^), central obese individuals may be at increased risk for DM due to excessive distribution of abdominal fat (18). People with normal weight central obesity have received little clinical attention. Guidelines from the American Obesity Society recommend routine monitoring of waist circumference (WC) to assess central obesity in overweight people, but do not recommend WC measurements with a normal BMI, as there is limited evidence that individuals with a normal BMI increase the risk associated with obesity (21). In addition, patients with normal-weight central obesity are often overlooked in developing risk reduction strategies. Due to the increasing incidence of central obesity over the years, individuals with normal-weight central obesity are common in the population, so assessing the health risks of these individuals is clinically important.

The aim of this study was to evaluate the association between different combinations of BMI and WC and the risk of DM, and to obtain quantitative data on the risk of normal-weight central obesity by using normal-weight without central obesity as a reference. Our hypothesis was that normal-weight central obesity increases the risk of developing DM compared to normal-weight without central obesity.

## 2. Methods

### 2.1. Study population

The study design of the National Health and Nutrition Examination Survey (NHANES) has been reported in detail in previous literature (22, 23). In short, NHANES is a cross-sectional survey conducted regularly by the Centers for Disease Control and Prevention (CDC) and the National Center for Health Statistics (NCHS), aimed at assessing the nutritional and health status of children and adults across the US. The study is conducted every 2 years and uses complex multi-stage sampling to ensure that the survey population is representative. The study design of the NHANES was approved by the review board of the NCHS, and all subjects provided informed consent at enrollment.

The study included participants from NHANES over five periods from 2009 to 2018. The criteria for inclusion are: (1) Age ≥ 18 years; (2) BMI ≥ 18.5 kg/m^2^; (3) Participants had available BMI and WC data; (4) The participants’ diabetes status was clear (yes or no). Finally, a total of 26,825 participants were included in the study.

### 2.2. Anthropometric measures

BMI is defined as weight divided by height squared, and its value is calculated in kg/m^2^. The weight and height were measured by trained NHANES personnel. The weight was measured using a portable electronic scale with an accuracy of 100 g and the height was measured using a tape measure with an accuracy of 0.1 cm. WC was measured in a standing position. The operator first palpated and located the bilateral iliac crest, placed the tape measure horizontally on the iliac crest, and gently attached it to the skin surface without compressing the skin. When the participant is in the minimum breathing stage, the data recorded by the tape measure is WC. Measurements of BMI and WC were recorded to the nearest 0.1 unit. BMI was divided into 3 categories: (1) normal weight (BMI: 18.5–24.9), overweight (BMI: 25–29.9), and obese (BMI ≥ 30.0) (24). The WC of central obesity was defined as ≥102 cm in males and ≥88 cm in females (25).

### 2.3. Ascertainment of diabetes mellitus

According to the latest guidelines from the American Diabetes Association (26), diabetes is considered to be present when: (1) Self-reported diabetes mellitus; (2) Receiving oral hypoglycemic medication or insulin therapy; (3) Fasting blood glucose level ≥ 126 mg/mL; (4) Oral glucose tolerance test 2-h blood glucose ≥200 mg/mL; (5) Glycosylated hemoglobin level ≥ 6.5%.

### 2.4. Other covariate assessments

Covariates include demographic characteristics (age, sex, race/ethnicity, education, marital status, and annual household income), smoking history, drinking history, hypertension status, and hyperlipidemia status. Smoking history included current smokers, never-smokers, and former smokers. Drinking history included never/mild drinkers, moderate drinkers, and heavy drinkers. Participants were considered to have hyperlipidemia if: Self-reported hyperlipidemia, taking lipid-lowering medications, total cholesterol ≥ 200 mg/mL, triglycerides ≥ 150 mg/mL, LDL ≥ 130 mg/mL, or HDL ≤ 40 mg/mL in male and ≤ 50 mg/mL in female (27). Participants were considered to have high blood pressure if: Self-reported hypertension, taking blood pressure lowering medications, systolic blood pressure ≥ 140 mmHg, or diastolic blood pressure ≥ 90 mmHg (28).

### 2.5. Statistical analysis

The overall goal of the study was to assess the effects of different categories of BMI and central obesity on the risk of DM. Therefore, a weighted logistic regression analysis was performed on 26,825 participants to obtain quantitative data on the risk of DM for different types of obesity. Since NHANES is a study with a complex stratified sampling design, corresponding weights were used for statistical analysis. For categorical data, weighted percentage (%) was used, whereas for continuous variables, weighted mean and 95% confidence interval (CI) were used. To investigate the relationship between different patterns of obesity and DM risk, we created a multivariate logistic regression model and adjusted for potential confounders, which included age, sex, race/ethnicity, etc.

We assessed whether these relationships varied by age (<50 vs. ≥50), race (nonwhite vs. white), education (under college vs. college or above), smoking status (never vs. ever), alcohol status (mild or never vs. heavy or moderate), hypertension status (yes vs. no), and hyperlipidemia status (yes vs. no). The association between BMI and WC is different in men and women (there may be statistical difference in combination) (29). Therefore, we also stratified the analysis by gender and adjusted for confounding factors. In addition, we plotted the receiver operator curve (ROC) and calculated the area under the curve (AUC) to assess the ability of WC and BMI to predict DM.

All statistics were performed using Stata, version 15.1 (Stata Corp. LP, Texas, United States). All tests were bilateral, and *p* < 0.05 was statistically significant.

## 3. Results

The average age of the 26,825 participants included in the study was 47 years old, and 690 (2.6%) of them were normal-weight central obese. Of the 26,825 participants, 7,740 (28.9%) had normal BMI, 8,786 (32.8%) were overweight and 10,299 (38.4%) were obese. According to the World Health Organization’s criteria for defining central obesity, 15,251 (56.9%) participants had central obesity. In contrast, when BMI was used as a measure of overweight or obesity, the detection rate of overweight and obesity was 71.1%, higher than the prevalence of central obesity (56.9%). Correlation analysis showed that WC was significantly correlated with BMI (correlation coefficient: 0.37, *p* < 0.001). Most of the participants were non-Hispanic white (65.1%), with education above high school (85.1%). The prevalence of hypertension and hyperlipidemia was 37.2 and 70.5%, respectively. In general, central obesity was associated with an increased prevalence of hypertension and hyperlipidemia compared with non-central obesity across the BMI categories. Baseline characteristics of participants in different combinations of BMI and WC can be obtained in Table 1.

**Table 1:** Baseline characteristics of the study population (*n* = 26,825) according to different combinations of BMI and WC.

	Normal Weight		Overweight		Obesity	
Variable	Normal WC	High WC	Normal WC	High WC	Normal WC	High WC
n. (male/female)	3,666/3,384	126/564	3,387/740	1,513/3,146	373/24	4,184/5,718
Age (Mean ± SD)	42.8 ± 19.2	56.6 ± 17.9	44.4 ± 17.1	54.6 ± 17.6	36.9 ± 14.5	49.3 ± 16.9
BMI (Mean ± SD)	22.2 ± 1.7	23.7 ± 1.0	26.9 ± 1.3	27.8 ± 1.4	31.2 ± 1.1	36.2 ± 5.8
WC (Mean ± SD)	81.9 ± 6.9	92.3 ± 4.0	93.8 ± 6.2	99.4 ± 6.8	97.7 ± 4.1	115.2 ± 12.9
**Race/ethnicity, n (%)**						
Mexican American	423 (6.0)	28 (4.1)	483 (11.7)	377 (8.1)	69 (17.4)	1,040 (10.5)
Other Hispanic	388 (5.5)	23 (3.4)	351 (8.5)	261 (5.6)	55 (13.9)	594 (6.0)
Non-Hispanic White	4,618 (65.5)	530 (76.8)	2,443 (59.2)	3,322 (71.3)	165 (41.6)	6,377 (64.4)
Non-Hispanic Black	691 (9.8)	43 (6.2)	458 (11.1)	382 (8.2)	69 (17.2)	1,386 (14.0)
Other race	931 (13.2)	66 (9.5)	392 (9.5)	317 (6.8)	39 (9.9)	505 (5.1)
**Education, n (%)**						
Under high school	924 (13.1)	99 (14.4)	669 (16.2)	731 (15.7)	64 (16.0)	1,505 (15.2)
High school or some college	3,426 (48.6)	354 (51.3)	1,981 (48.0)	2,526 (54.2)	232 (58.5)	6,050 (61.1)
College graduate or above	2,700 (38.3)	237 (34.3)	1,477 (35.8)	1,402 (30.1)	101 (25.5)	2,347 (23.7)
**Income, n (%) $**						
<20,000	1,241 (17.6)	113 (16.4)	582 (14.1)	727 (15.6)	59 (14.8)	1,604 (16.2)
20,000–44,999	1,657 (23.5)	173 (25.1)	978 (23.7)	1,169 (25.1)	85 (21.3)	2,733 (27.6)
>45,000	4,152 (58.9)	404 (58.5)	2,567 (62.2)	2,763 (59.3)	253 (63.9)	5,565 (56.2)
**Marital status, n (%)**						
Live with someone	4,117 (58.4)	438 (63.5)	2,823 (68.4)	3,117 (66.9)	256 (64.5)	6,258 (63.2)
Live alone	2,933 (41.6)	252 (36.5)	1,304 (31.6)	1,542 (33.1)	141 (35.5)	3,644 (36.8)
**Smoking, n (%)**						
Never	4,103 (58.2)	376 (54.5)	2,332 (56.5)	2,567 (55.1)	227 (57.1)	5,486 (55.4)
Former	1,671 (23.7)	198 (28.7)	1,209 (29.3)	1,435 (30.8)	126 (31.7)	3,089 (31.2)
Current	1,276 (18.1)	116 (16.8)	586 (14.2)	657 (14.1)	44 (11.2)	1,327 (13.4)
**Drinking, n (%)**						
Mild or never	2,115 (30.0)	227 (32.9)	1,160 (28.1)	1,715 (36.8)	110 (27.6)	4,198 (42.4)
Moderate	1,918 (27.2)	164 (23.7)	1,193 (28.9)	1,183 (25.4)	134 (33.7)	2,892 (29.2)
Heavy	3,017 (42.8)	399 (43.4)	1,775 (43.0)	1,761 (37.8)	153 (38.7)	2,812 (28.4)
**Hypertension, n (%)**						
No	5,499 (78.0)	424 (61.4)	2,967 (71.9)	2,642 (56.7)	272 (68.4)	4,911 (49.6)
Yes	1,410 (20.0)	266 (38.6)	1,160 (28.1)	2,017 (43.3)	125 (31.6)	4,991 (50.4)
**Hyperlipidaemia, n (%)**						
No	3,455 (49.0)	167 (24.2)	1,292 (31.3)	992 (21.3)	140 (35.3)	1,881 (19.0)
Yes	3,596 (51.0)	523 (75.8)	2,835 (68.7)	3,667 (78.7)	257 (64.7)	8,021 (81.0)

Continuous variables are expressed by means and standard deviation (SD), categorical variables are expressed in numbers (percentages).

Body mass index calculated as weight in kilograms divided by height in meters squared and categorized as normal weight (18.5–24.9), overweight (25.0–29.9), and obesity (30.0). Waist circumference categorized as normal (≤88 cm) and high (>88 cm) for women, as normal (≤102 cm) and high (>102 cm) for men.

WC, Waist circumference; BMI, Body mass index.

Table 2 shows the association between different combinations of BMI and WC and the risk of DM. Participants with central obesity in each BMI category had a higher risk of DM than participants with normal-weight central obesity, while participants with no central obesity in the overweight or obese category had a slightly higher or lower risk of DM (OR: 1.62, 95% CI: 1.33–1.98; OR: 0.81, 95% CI: 0.48–1.34, respectively). Similar results were observed after adjusting for confounding factors such as demographics, smoking and alcohol consumption. When stratified by gender, it was found that men with normal-weight central obesity and obesity and central obesity had a similar risk of DM (OR: 3.83, 95% CI: 2.10–5.97; OR: 4.20, 95% CI: 3.48–5.08, respectively), and the risk was higher than that of men with any combination of BMI and WC, including men with overweight and no central obesity (OR: 3.39, 95% CI: 2.68–4.28) and men with obesity and no central obesity (OR: 0.53, 95% CI: 0.30–0.91). Compared with normal-weight non central obesity, obese non central obesity men (*n* = 373) have a decreased risk of diabetes (OR: 0.53, 95% CI: 0.30–0.91), while obese non central obesity women (*n* = 24) have an increased risk of diabetes (OR: 2.39, 95% CI: 0.47–12.11).

**Table 2:** Association of different combinations of BMI and WC status with diabetes mellitus among 26,825 participants.

	Normal Weight		Overweight		Obesity	
Model	Normal WC	High WC	Normal WC	High WC	Normal WC	High WC
**Total**						
Model 1	1 (Reference)	2.37 (1.75–3.23)	1.62 (1.33–1.98)	3.36 (2.85–3.97)	0.81 (0.48–1.34)	5.85 (5.06–6.76)
Model 2	1 (Reference)	2.21 (1.62–3.01)	1.59 (1.30–1.95)	3.02 (2.55–3.57)	0.78 (0.46–1.30)	5.19 (4.47–6.03)
Model 3	1 (Reference)	1.57 (1.13–2.18)	1.31 (1.07–1.62)	2.02 (1.69–2.41)	0.60 (0.35–1.02)	3.25 (2.78–3.80)
**Male**						
Model 1	1 (Reference)	3.83 (2.10–5.97)	1.21 (0.96–1.51)	3.39 (2.68–4.28)	0.53 (0.30–0.91)	4.20 (3.48–5.08)
Model 2	1 (Reference)	3.61 (2.03–5.66)	1.19 (0.94–1.50)	3.09 (2.42–3.95)	0.51 (0.29–0.87)	3.96 (3.25–4.82)
Model 3	1 (Reference)	2.25 (1.57–3.86)	0.94 (0.74–1.19)	1.86 (1.44–2.40)	0.37 (0.21–0.66)	2.37 (1.93–2.92)
**Female**						
Model 1	1 (Reference)	3.89 (2.72–5.57)	1.69 (0.96–3.00)	4.53 (3.52–5.83)	2.39 (0.47–12.11)	9.57 (7.57–12.10)
Model 2	1 (Reference)	3.63 (2.51–5.24)	1.62 (0.90–2.89)	3.98 (3.07–5.15)	2.74 (0.55–13.72)	7.94 (6.22–10.14)
Model 3	1 (Reference)	2.64 (1.80–3.87)	1.54 (0.84–2.80)	2.86 (2.19–3.73)	3.05 (0.62–14.88)	5.22 (4.06–6.72)

The data were expressed with odds ratios (OR) and corresponding 95% confidence intervals (CI).

Body mass index calculated as weight in kilograms divided by height in meters squared and categorized as normal weight (18.5–24.9), overweight (25.0–29.9), and obesity (30.0). Waist circumference categorized as normal (≤88 cm) and high (>88 cm) for women, as normal (≤102 cm) and high (>102 cm) for men.

Model 1: No covariates were adjusted; Model 2: Age, race/ethnicity, education, smoking and drinking status were adjusted; Model 3: Model 2 + hypertension and hyperlipidemia status.

WC, Waist circumference; BMI, Body mass index.

**Figure 1:**
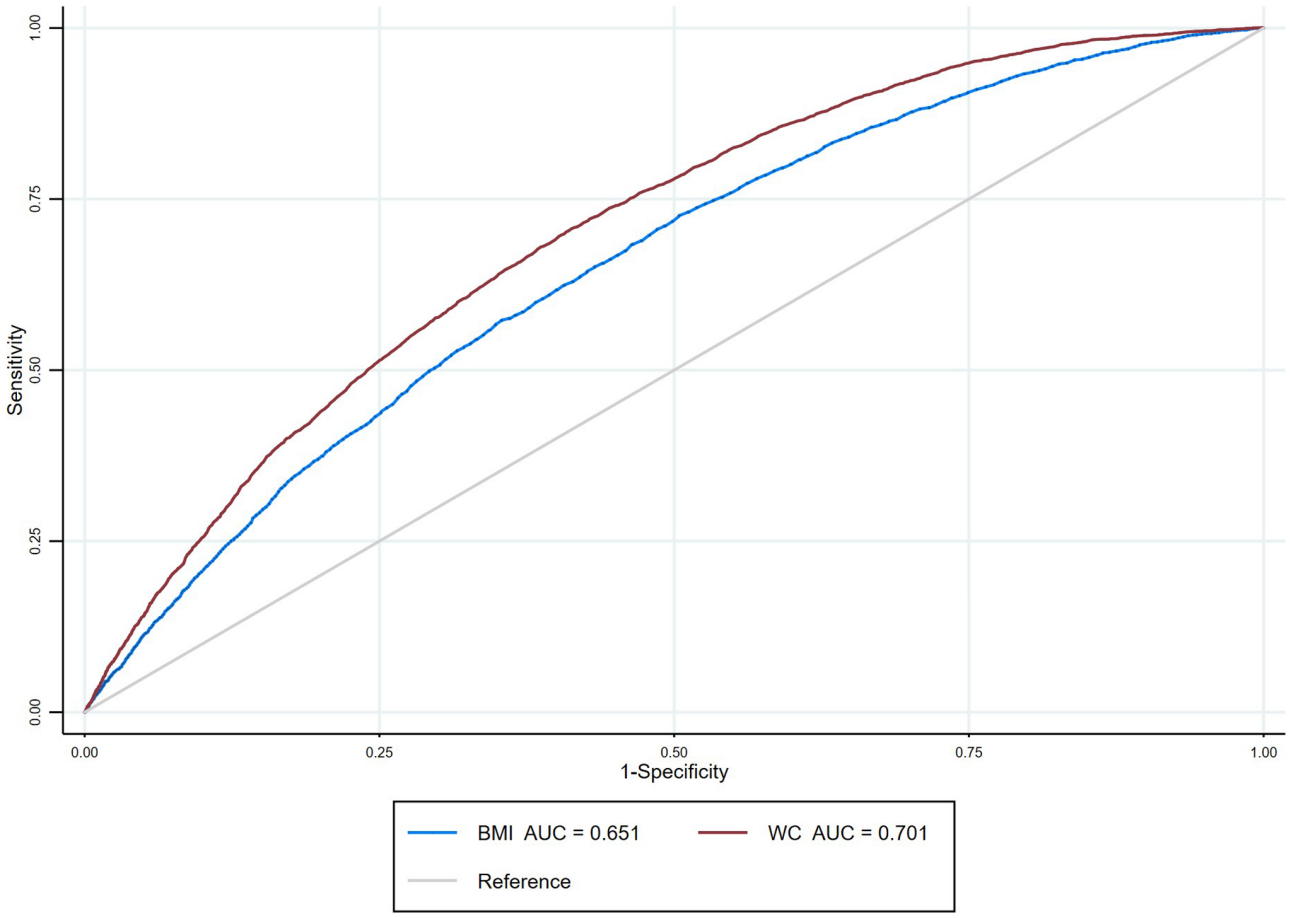
Receiver operating characteristic curves for BMI and waist circumference (WC) predicting diabetes.

Table 3 shows the results of different combinations of BMI and WC and the risk of DM after stratification by age, race/ethnicity, education level, smoking status, etc. Participants with different levels of education had similar risks of developing DM. Participants with a history of hyperlipidemia, hypertension, and older participants (age ≥ 50) may be at decreased risk of developing DM. Participants with heavy and moderate alcohol consumption had a higher risk of DM than those with mild or no alcohol consumption. In addition, ROC was plotted to assess the ability of BMI and WC to predict DM (Figure 1). The results showed that, compared with BMI, WC had a larger AUC; (AUC: 0.651, 95% CI: 0.643–0.660; AUC: 0.701, 95% CI: 0.693–0.709, respectively), which may be a better predictor of DM. When stratified by gender, WC showed a larger AUC for both male and female participants (Table 4).

**Table 3:** Stratified analyses for the association of BMI and WC status with diabetes mellitus.

	Normal Weight		Overweight		Obesity	
Characteristic	Normal WC	High WC	Normal WC	High WC	Normal WC	High WC
**Age**						
<50	1 (Reference)	2.57 (1.29–5.09)	1.92 (1.38–2.67)	3.66 (2.69–4.98)	1.52 (0.78–2.97)	8.05 (6.29–10.30)
≥50	1 (Reference)	1.30 (0.91–1.86)	1.69 (1.30–2.21)	2.00 (1.63–2.47)	1.22 (0.52–2.86)	4.05 (3.35–4.91)
**Race**						
White	1 (Reference)	2.31 (1.45–3.67)	1.93 (1.36–2.74)	3.97 (3.01–5.22)	0.76 (0.222.58)	8.08 (6.29–10.37)
Nonwhite	1 (Reference)	3.26 (2.24–4.74)	1.26 (1.06–1.51)	3.00 (2.53–3.55)	0.65 (0.39–1.07)	3.75 (3.25–4.31)
**Education**						
Under college	1 (Reference)	2.20 (1.57–3.08)	1.54 (1.23–1.92)	2.95 (2.46–3.55)	0.76 (0.43–1.35)	5.01 (4.25–5.89)
College or above	1 (Reference)	2.11 (1.01–4.38)	1.61 (1.04–2.50)	3.53 (2.43–5.13)	0.52 (0.15–1.76)	6.31 (4.57–8.71)
**Smoking status**						
Never smoked	1 (Reference)	2.95 (1.96–4.42)	1.44 (1.09–1.90)	3.44 (2.74–4.32)	0.75 (0.38–1.46)	5.79 (4.73–7.10)
Ever smoked	1 (Reference)	1.81 (1.13–2.89)	1.80 (1.36–2.39)	3.24 (2.54–4.12)	0.86 (0.40–1.84)	5.84 (4.74–7.20)
**Drinking status**						
Mild or never	1 (Reference)	2.06 (1.23–3.44)	1.46 (0.99–2.16)	2.71 (1.99–3.68)	0.56 (0.24–1.29)	4.78 (3.63–6.30)
Heavy or moderate	1 (Reference)	3.66 (2.06–6.53)	2.47 (1.79–3.40)	4.54 (3.36–6.12)	0.93 (0.33–2.64)	7.94 (6.17–10.21)
**Hypertension**						
No	1 (Reference)	2.51 (1.43–4.42)	1.56 (1.13–2.14)	2.86 (2.18–3.76)	1.00 (0.43–2.30)	4.80 (3.80–6.07)
Yes	1 (Reference)	1.38 (0.96–1.99)	1.28 (0.98–1.67)	2.05 (1.65–2.56)	0.46 (0.24–0.87)	3.31 (2.72–4.02)
**Hyperlipidaemia**						
No	1 (Reference)	2.32 (1.14–4.74)	1.88 (1.16–3.06)	4.61 (3.21–6.62)	1.30 (0.46–3.68)	7.08 (5.22–9.59)
Yes	1 (Reference)	1.79 (1.27–2.51)	1.27 (1.02–1.58)	2.37 (1.96–2.86)	0.60 (0.33–1.08)	4.14 (3.50–4.89)

The data were expressed with odds ratios (OR) and corresponding 95% confidence intervals (CI).

Body mass index calculated as weight in kilograms divided by height in meters squared and categorized as normal weight (18.5–24.9), overweight (25.0–29.9), and obesity (30.0). Waist circumference categorized as normal (≤88 cm) and high (>88 cm) for women, as normal (≤102 cm) and high (>102 cm) for men.

WC, Waist circumference; BMI, Body mass index.

**Table 4:** The area under curve of BMI and WC in the prediction of DM.

Characteristic	BMI	*p*	WC	*p*
Total	0.651 (0.643–0.660)	<0.001	0.701 (0.693–0.709)	<0.001
Male	0.635 (0.623–0.647)	<0.001	0.691 (0.679–0.702)	<0.001
Female	0.669 (0.658–0.681)	<0.001	0.709 (0.699–0.721)	<0.001

The data were expressed with area under curve (AUC) and corresponding 95% confidence intervals (CI).

BMI: body mass index; WC: waist circumference.

## 4. Discussion

The results of this large cross-sectional study, which included 26,825 participants from 2009 to 2018, showed that participants with normal-weight central obesity were at an increased risk of developing DM. When stratified by gender, it was found that men with normal-weight central obesity and obesity and central obesity had a similar risk of DM, and the risk was higher than that of men with any combination of BMI and WC, including men with overweight and no central obesity and men with obesity and no central obesity. In addition, the results of the receiver’s operator curve suggest that WC may be a better predictor of DM than BMI.

Some previous literature has reported the association between WC and BMI and the risk of DM, but few studies have investigated the risk of DM in normal-weight central obesity. A cohort study of 3,001 previously diabetic-free participants reported that both WC and BMI were related to an increased risk of type 2 diabetes (T2D) after a median follow-up of 4.7 years, and that BMI was more effective than WC in predicting the occurrence of T2D (30). This is consistent with the conclusions of another observational study involving 9,962 elderly people, which showed that among five different anthropometric parameters such as BMI and WC, BMI was the strongest predictor of the occurrence of DM (31). Another study showed that WC and WHtR were stronger predictors of DM than BMI (32). However, the BMI and WC classifications of these studies were based on baseline conditions and did not take into account changes in body size during follow-up. Studies have shown that with age, the distribution of body fat can change correspondingly, mainly manifested as the accumulation of abdominal fat and the reduction of subcutaneous fat, and these changes can be independent of weight gain (33). Thus, these studies may have a misclassification bias associated with changes in body weight and waist circumference during follow-up, which may affect the stability of the results. In situations where the weight status is mostly defined by BMI, few studies have focused on the population of normal-weight central obesity. Recent literatures have suggested that normal-weight central obesity may have an increased risk of disease, but the outcome measures were defined as cancer and cardiovascular death events (29, 34). Our results are similar to those of a recent cross-sectional study in Japan, which showed an increased risk of DM among 117,163 participants with normal-weight central obesity (19). However, the study did not conduct a stratified analysis of gender. Published evidence shows that the pattern of relationship between WC and BMI is significantly different in males and females (29).

The following reasons may explain why participants with normal-weight central obesity have an increased risk of DM. Firstly, the accumulation of visceral adipose tissue may be an explanation. The accumulation of visceral adipose tissue may have implications for the development of obesity-related diseases, such as diabetes, cardiovascular disease, and some cancers (35–38). And visceral fat accumulation often manifests as abdominal obesity, which is the most common feature of metabolic disease (35). Our linear regression results show that WC and BMI have a weak correlation (correlation coefficient: 0.37), which may prove that WC and BMI provide different information. In general, WC is a measure of abdominal fat accumulation, while BMI is a measure of fat content (20). Second, patients with normal-weight central obesity may have less muscle mass, which is associated with improved metabolic status (20). Previous studies have shown that decreased muscle tissue content may be associated with adverse health outcomes (39–41). Thirdly, gluteo-femoral adipose tissue has a protective effect on metabolism, which can be transmitted through beneficial adipokines such as leptin and adiponectin (42). In individuals with normal-weight central obesity, excess accumulation of abdominal fat may lead to loss of protective gluteo-femoral adipose tissue, which may increase the risk of DM. Conversely, overweight or obese patients may have more gluteal-femoral adipose tissue, which may partly explain why obese participants without central obesity may have a lower incidence of DM. Fourthly, the concept of hepatokines was recently proposed (43). Hepatokines are proteins secreted by the liver that regulate glucose and lipid metabolism (44). The accumulation of fat in the liver affects metabolism by regulating the secretion of specific hepatokines. A Mendelian randomization showed that increased liver fat content was associated with a higher risk of type 2 diabetes (45).

Our conclusions have important public health and clinical value. First, in the current guidelines, BMI is still the standard index to measure obesity clinically, and individuals with normal BMI are classified as normal in practice regardless of their WC. In the recent joint guidelines for obesity management from the American College of Cardiology, the American Heart Association, and the Obesity Society, waist measurement is recommended for individuals who are overweight or class I obesity to assess the risk of obesity-related comorbidities, and not for individuals with a normal BMI because there is insufficient evidence that such individuals increase the risk of obesity (46). The guidelines may convey to clinicians and the general public that individuals with a normal BMI do not have any risk associated with obesity. Whereas individuals with central obesity, even with a normal BMI, are at an increased risk of developing DM. Second, assessing the distribution of adipose tissue usually requires specialized medical equipment, such as magnetic resonance imaging (MRI), and computed tomography (CT). Compared with these equipment, anthropometric parameters such as BMI, WC and WHtR are convenient and easy to obtain, which can greatly improve patient engagement. Our results suggest that WC is a stronger predictor of diabetes than BMI. Therefore, when developing strategies to reduce diabetes risk, such as early exercise and diet modification and timely medical intervention, more attention may be paid to changes in WC. In people with normal-weight central obesity, regular exercise, even with little or no weight loss, can bring about a variety of beneficial changes, such as improved glucose homeostasis and insulin sensitivity (11, 47). In addition, in people with normal-weight central obesity, caloric restriction shows good promise in reducing waist circumference and improving metabolism (48).

### 4.1. Strengths and limitations

To the best of our knowledge, this is the first cross-sectional study to comprehensively assess the association between different types of obesity and the risk of DM. We used nationally representative standardized data from the NHANES database, which increased the stability of our results. In addition, WC is a simple and reliable measure of visceral fat accumulation, which increases the general applicability of our results. However, our study has the following limitations: First, due to the cross-sectional design of this study, a causal link between obesity and DM cannot be inferred. Second, some of the participants’ diabetes diagnoses were based on self-reporting, which may lead to recall bias. Third, the sample size of obese women with no central obesity is small, which may affect the stability of our results to some extent. In general, a larger BMI is often associated with a larger WC, so obesity without central obesity is not common in the population. The number of obese women with no central obesity included in the analysis was only 24, so the results have a relatively wide 95% CI and must be interpreted with caution. In future studies, more attention can be paid to the obese without central obesity population to investigate their risk of developing metabolic diseases.

## 5. Conclusion

The results of our cross-sectional study showed that participants with central obesity of normal weight had a higher risk of developing DM. When stratified by sex, men with normal-weight central obesity, obesity and central obesity were found to have a similar risk of DM and an increased risk than men with any combination of BMI and WC, including men who were overweight with no central obesity and obese with no central obesity. Our results highlight that BMI alone cannot distinguish the distribution of adipose tissue, and that even in people with normal BMI, the risk of DM may be increased due to excessive accumulation of abdominal fat. The exact mechanism between different types of obesity and a higher risk of DM needs to be clarified with larger sample sizes and more well-designed prospective studies.

## Data availability statement

The original contributions presented in the study are included in the article/supplementary material, further inquiries can be directed to the corresponding author.

## Ethics statement

Research involving human participants were approved by the National Center for Health Statistics (NCHS) Research Ethics Review Board. The participants provided written informed consent to participate in this study.

## Author contributions

XSJ designed the study, analyzed the feasibility of the study, and wrote the manuscript of the paper. QYC, JHL, GFW, and LHL extracted the data. SJ, XZ, and ZQL collated and merged the data. JJL conducted the statistical analysis, drew figures and tables. ACY reviewed the manuscript, provided critical scientific input, and checked the whole process of the study. All authors contributed to the article and approved the submitted version.

## Conflict of interest

The authors declare that the research was conducted in the absence of any commercial or financial relationships that could be construed as a potential conflict of interest.

## Publisher’s note

All claims expressed in this article are solely those of the authors and do not necessarily represent those of their affiliated organizations, or those of the publisher, the editors and the reviewers. Any product that may be evaluated in this article, or claim that may be made by its manufacturer, is not guaranteed or endorsed by the publisher.
